# Supplementary material for: MetaRibo-Seq measures translation in microbiomes
Source: Nat Commun. 2020 Jun 29;11:3268. doi: 10.1038/s41467-020-17081-z (PMC7324362; doi:10.1038/s41467-020-17081-z)
Supplement: Supplementary file 10 — Supplementary Data 7 [file 41467_2020_17081_MOESM10_ESM.zip › File2/Confidence_VeryHigh_Taxonomy/110232_out.krona.html]

Javascript must be enabled to view this page.

members
magnitude
magnitudeUnassigned
count
unassigned
taxon
rank

110232\_out

29

29
2
superkingdom

29
1239
phylum

186801
class
29

29
186802
order

29
family
186806

genus
1730
29

7

SRS013951\_contig\_number\_2316SRS014235\_contig\_number\_40110SRS015578\_contig\_number\_26692SRS015854\_contig\_number\_contig-100\_3860.106880SRS098073\_contig\_number\_4773SRS1055022\_contig\_number\_contig-100\_826.141569SRS142781\_contig\_number\_11777
1897001
species

1262895
species

SRS014287\_contig\_number\_15937SRS062427\_contig\_number\_4246SRS063985\_contig\_number\_8174SRS075773\_contig\_number\_23442SRS104400\_contig\_number\_55849SRS104975\_contig\_number\_19836SRS147346\_contig\_number\_42473SRS893285\_contig\_number\_contig-100\_387.23410
8

14

SRS014855\_contig\_number\_1011SRS015431\_contig\_number\_81531SRS016629\_contig\_number\_contig-100\_7985.7986SRS018623\_contig\_number\_contig-100\_788.48311SRS019068\_contig\_number\_50386SRS019445\_contig\_number\_contig-100\_14978.62143SRS022609\_contig\_number\_contig-100\_9762.51799SRS045244\_contig\_number\_2189SRS051031\_contig\_number\_contig-100\_16371.58935SRS052697\_contig\_number\_24717SRS063040\_contig\_number\_1382SRS065504\_contig\_number\_4735SRS104311\_contig\_number\_43236SRS148784\_contig\_number\_17669
species
142586
